# New insights of karyoevolution in the Amazonian turtles *Podocnemis expansa* and *Podocnemis unifilis* (Testudines, Podocnemidae)

**DOI:** 10.1186/s13039-016-0281-5

**Published:** 2016-09-27

**Authors:** R. C. R. Noronha, L. M. R. Barros, R. E. F. Araújo, D. F. Marques, C. Y. Nagamachi, C. Martins, J. C. Pieczarka

**Affiliations:** 1 Laboratório de Citogenética, Instituto de Ciências Biológicas, Universidade Federal do Pará, Rua Augusto Corrêa, 01 - Guamá, 66075-110 Belém, PA Brazil; 2 Laboratório Genômica Integrativa, Universidade Estadual Paulista “Julio de Mesquita Filho”, Botucatu, SP Brazil; 3 CNPq Researcher, Belém, Pará Brazil

**Keywords:** Biodiversity, Molecular markers, Evolution, Turtles

## Abstract

**Background:**

Cytogenetic studies were conducted in the Brazilian Amazon turtles, *Podocnemis expansa* Schweigger, 1912 (PEX) and *Podocnemis unifilis* Troschel, 1848 (PUN) to understand their karyoevolution. Their chromosomal complements were compared using banding techniques (C, G-, Ag-NOR and Chromomycin A_3_) and fluorescence in situ hybridization (FISH), and efforts were made to establish evolutionary chromosomal relationships within the Podocnemidae family.

**Results:**

Our results revealed that both species have a chromosome complement of 2n = 28. For PEX and PUN, the fundamental numbers (FNs) were 54 and 52, respectively and the karyotypic formulas (KFs) were 24 m/sm + 2st + 2a and 22 m/sm + 2st + 4a, respectively. G-banding evidenced homologies between the two species and allowed identify a heteromorphic pair (chromosome pair 10) in PUN. In PEX, constitutive heterochromatin (CH) was found in the centromeric regions of pairs 1, 2, 4, 6 and 11 and on 9p. In PUN, CH was observed in the centromeric regions of all chromosomes, and in small proximal bands on 1p, 2p, 3q, 4q, 5q, 9q, 10q and 11q. Moreover, CH amplification was seen in one of the homologs of pair 10 (the heteromorphic pair). The CMA3 staining results were consistent with the CH findings. Ag-NOR staining showed that nucleolar organizing regions (NORs) were localized in the pericentromeric region of pair 1 in both species, and this result was confirmed by the 18S rDNA FISH probe. FISH with telomeric probes identified telomeric sequences in the distal regions of all chromosomes. In addition, interstitial telomeric sequences (ITSs) were present in seven chromosome pairs of PUN, perhaps reflecting the amplification of telomere-like sequences. FISH with a probe against the transposable element (TE), *Rex 6*, revealed that it is dispersed in euchromatic regions of the first chromosome pairs of both species. This is the first report describing the FISH-based analysis of PEX and PUN for the 18S rDNA, *Rex 6* and human telomeric sequences.

**Conclusions:**

Our results contribute to clarifying the chromosomal homologies and rearrangement mechanisms that occurred during the evolution of these species, and may help researchers uncover new markers that will improve our understanding of the taxonomy and systematic classification of Podocnemidae.

**Trial registration:**

ISRCTN ISRCTN73824458. Registered 28 September 2014. Retrospectively registered.

## Background

The order Testudines is regarded as one of the oldest lineages of vertebrates [12, 15]. This group, characterized by slow growth, delayed sexual maturity and long lifespans [29], currently comprises 12 families and about 285 species [30].

Testudines are recognized by three different karyotypes groups: I) karyotypes with high diploid numbers, 2n = 60–64 chromosomes, with the presence of microchromosomes; II) karyotypes with diploid numbers varying from 2n = 50–56 chromosomes and less microchromosomes than the first group; III) karyotypes with low diploid numbers, ranging from 2n = 26–28 chromosomes, and without microchromosomes [2, 3, 7, 8]. The Podocnemidae family belongs to the last karyotype group. Cytogenetic analyses have contributed significantly to the characterization of taxa and evolutionary relationship, as they allow studies that infer the chromosomal evolution occurred between and within taxa [9].

The Podocnemidae family comprises two genera (*Podocnemis* and *Peltocephalus*) whose members are distributed throughout South America and are readily found in the Amazonian region [24]. The species of this family include *Podocnemis expansa* (Schweigger, 1912), *Podocnemis sextuberculata* (Cornalina, 1849), *Podocnemis unifilis* (Troschel, 1848), *Podocnemis erythrocephala* (Spix, 1824) and the single species of the second genus, *Peltocephalus dumerilianus* (Schweigger, 1812; [4]).

According to Vargas-Ramirez et al. [41], phylogenetic analysis with molecular data (mitochondrial and nuclear) support the monophyly of the Podocnemidae family (*Erymmochelys, Peltocephalus and Podocnemis*) suggesting that vicariance events were responsible for the diversification of the* Podocnemis* species, classifying the P. *expansa* species in the basal position relative to *P*. *unifilis*.

The cytogenetic studies carried out in *Podocnemis* to date have been limited to the gross morphological characterization of chromosomes, the localization of the nucleolar organizing regions (NORs) [2, 8, 13, 22] and, more recently, G- and C-banding [18], confirming the karyotipic conservatism within the genus [6]. Molecular cytogenetic analysis can be a useful tool to investigate mechanisms by which this group has evolved, as well as to elucidate the karyotype homologies of the species.

According to Feschotte and Phrithan [16] the movement of transposable elements can promote structural changes. This could lead to events such as chromosomal rearrangements, changes in patterns of gene regulation, and promote genetic variability. Consequently, it plays a key role in the evolution of genes and genomic structure of eukaryotes, thus generating biological innovations.

Among the retrotransposons, members of the *Rex* family (e.g., *Rex 1*, *Rex 3* and *Rex* 6) seem to be rather abundant in Teleostei [33, 44]. However, they have not previously been analyzed in any species of Testudines.

In the present study, we compared the karyotypes of PEX and PUN to clarify the rearrangements involved in the karyotypic differentiation of *Podocnemis*. Furthermore, cytogenetic analysis may improve our understanding of the mechanisms through which this group evolved, such as by revealing the karyotypic homologies of these species.

## Results

### Classical cytogenetics

**Fig. 1:**
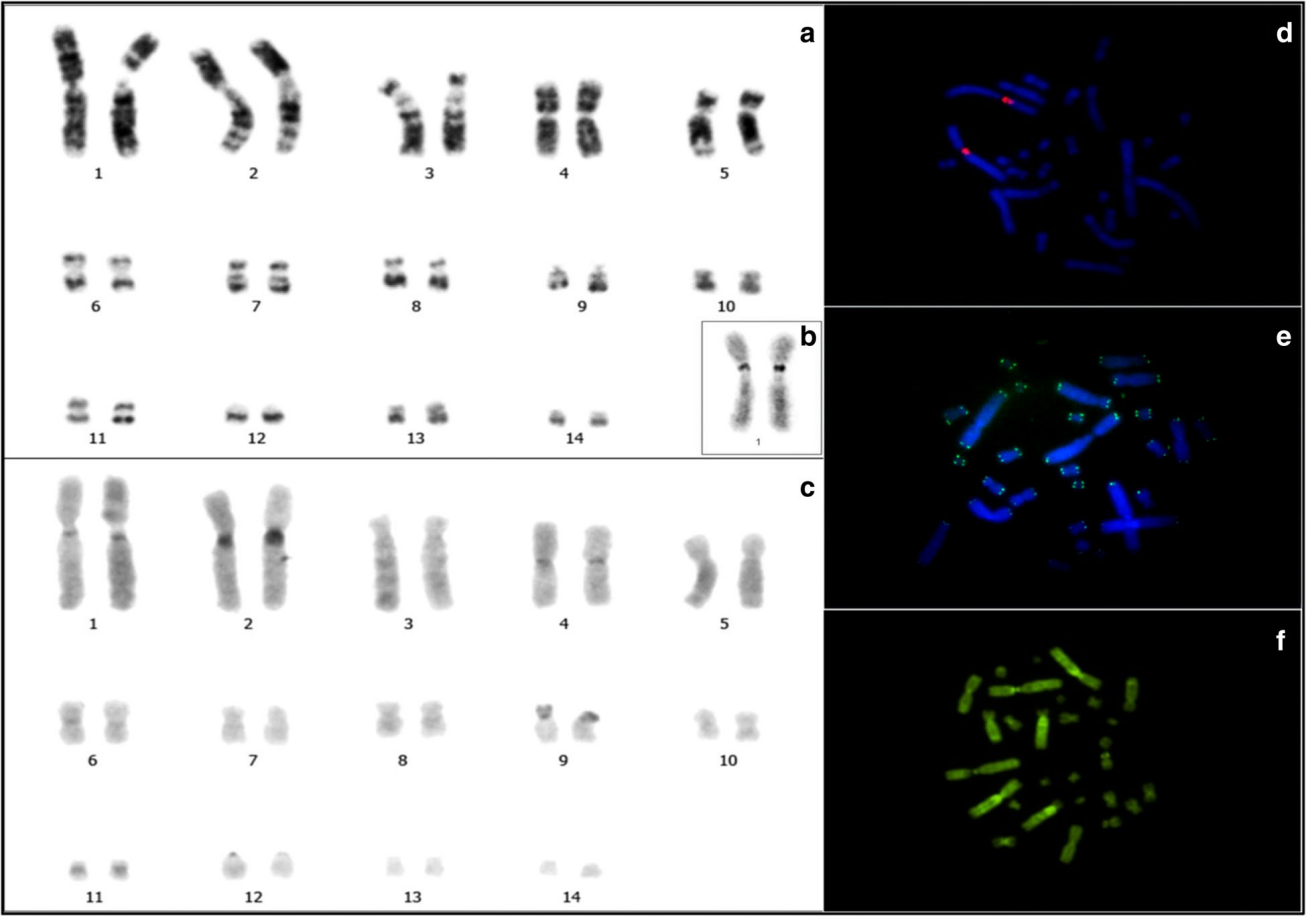
*Podocnemis expansa* (PEX). **a** G-banding. **b** Ag-NOR. **c** C-banding. **d** FISH human telomeric probes. **e** FISH 18S rDNA probes. **f** CMA3. Bar: 5 μm

**Fig. 2:**
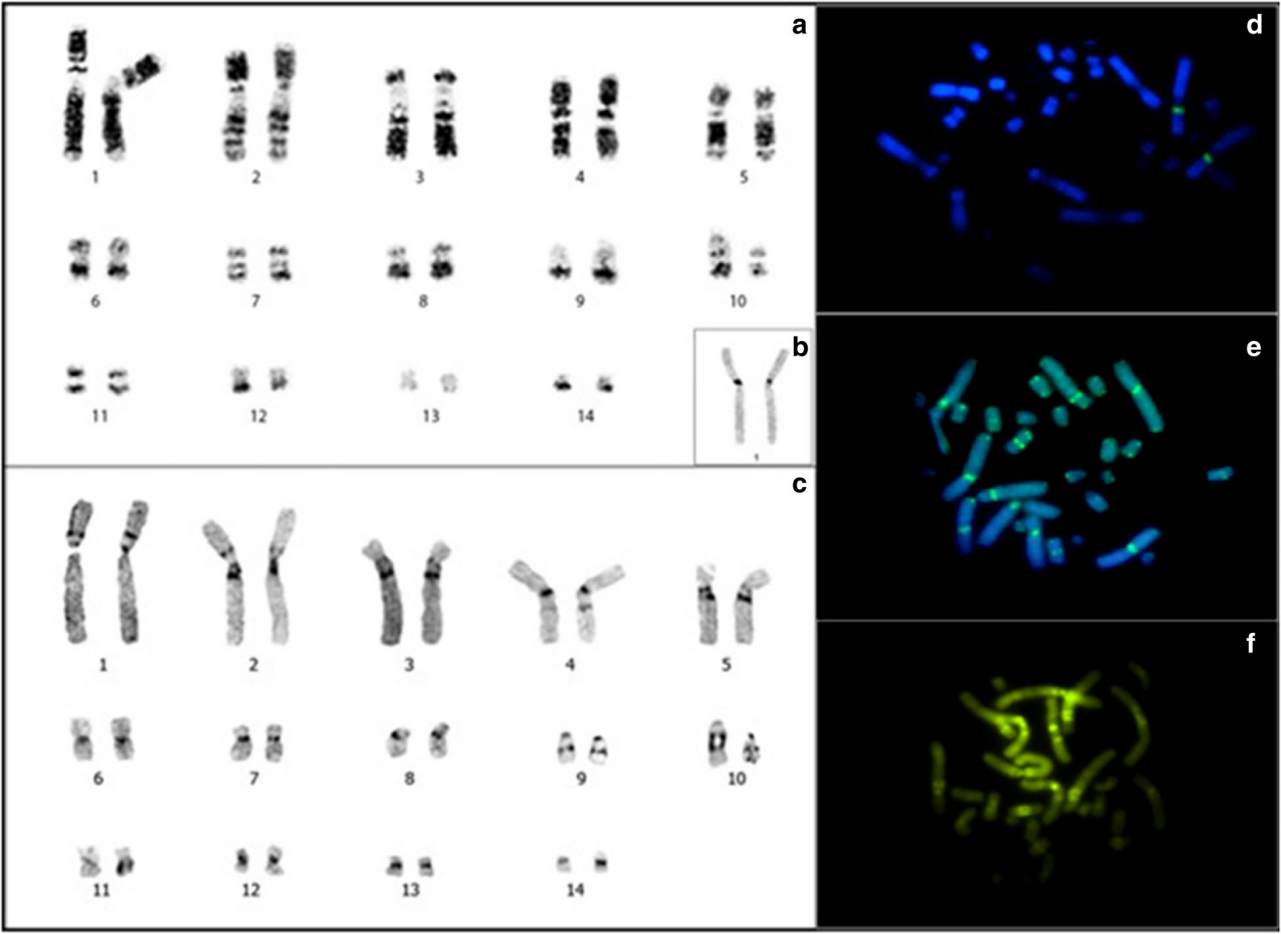
*Podocnemis unifilis* (PUN). **a** G-banding. **b** Ag-NOR. **c** C-banding. **d** FISH human telomeric probes. **e** FISH 18S rDNA probes. **f** CMA3. Bar: 5 μm

PEX and PUN have conserved diploid numbers of 2n = 28. The fundamental numbers (FNs) of these species are 54 and 52, respectively. The karyotype formula (KF) of PEX (Fig. 1a) consist of 24 metacentric and submetacentric chromosomes, two subtelocentric chromosomes and two acrocentric chromosomes, while that of PUN (Fig. 2a) consisted of 22 metacentric and submetacentric chromosomes, two subtelocentric chromosomes and four acrocentric chromosomes (Table 1). No sex chromosome differentiated was found.

**Table 1:** Karyotype data of the studied species

Species	2n	NF	KF			
			M (pairs)	SM (Pairs)	ST (Pairs)	A (Pairs)
PEX	28	54	12 (1,4,6,10,11,14)	12 (2,5,7,8,9,13)	2 (3)	2 (12)
PUN	28	52	12 (1,4,6,10,11,14)	10 (2,5,7,8,13)	2 (3)	4 (9,10)

G-banding revealed homologies between species and the presence of a heteromorphic pair 10 in PUN (Fig. 2a). In PEX, C-banding showed constitutive heterochromatin (CH) in the pericentromeric regions of pairs 1, 2, 4, 6, 10 and 11, as well as labeling all over the short arm and centromere of pair 9 (Fig. 1c). In PUN, CH was observed in the centromeric regions of all chromosomes, as small proximal bands on the short arms of pairs 1 and 2, and on the long arms of pairs 3, 4, 5, 9 and 10 (Fig. 2c). Staining with the GC-specific fluorochrome, Chromomycin A3 (CMA3) indicated the presence of CH regions in both PEX and PUN (Figs. 1f and 2f, respectively). Both species showed simple NORs located at the secondary constriction of the short arm of pair 1, flanked by centromeric C-banding- and CMA3-positive staining (Figs. 1b and 2b).

### Molecular cytogenetics

**Fig. 3:**
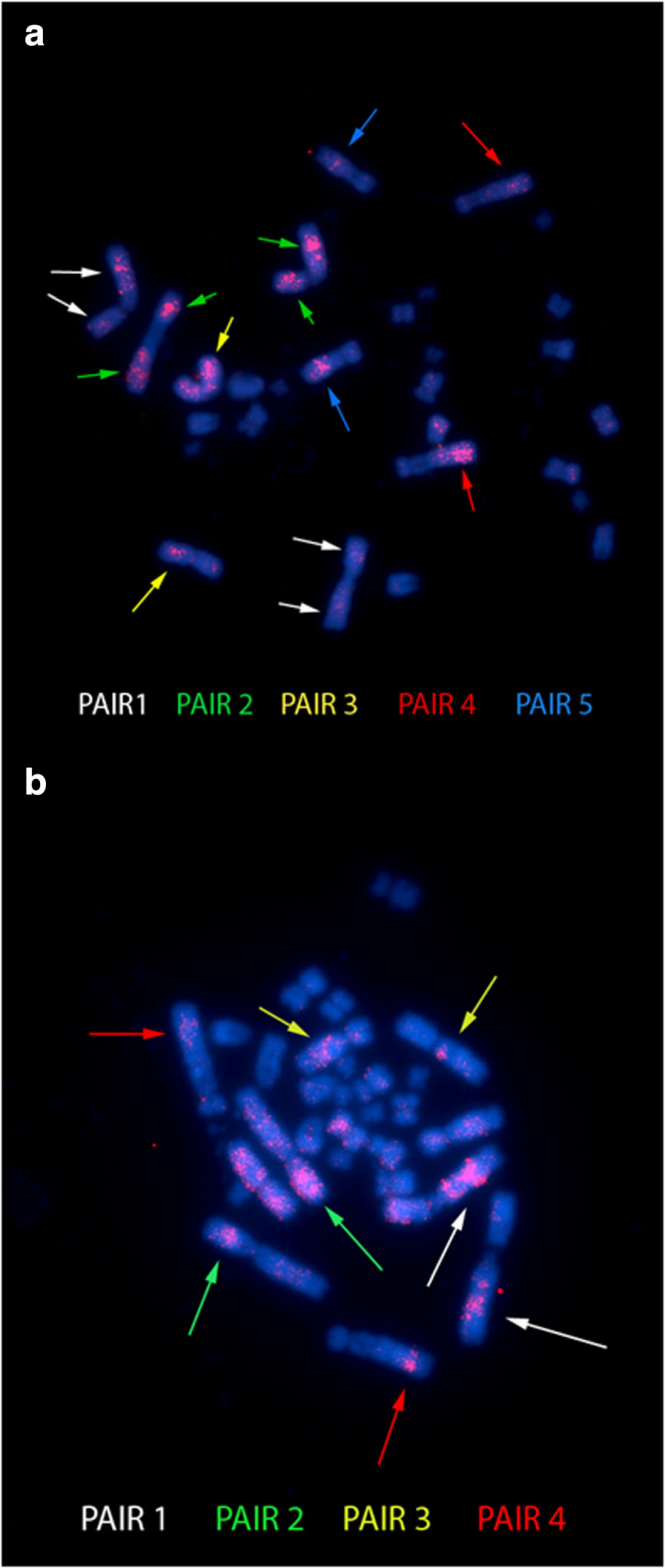
**a** and **b** Metaphases of *Podocnemis expansa* (PEX) and *P. unifilis* (PUN) probed with *Rex 6* transposons. Bar: 5 μm

FISH with the 18S rDNA probe yielded hybridization to a single site in each species, located at the secondary constriction of the short arm of pair 1agreed with NORs (Figs. 1d and 2d). FISH with telomeric probes revealed that such sequences were present in the distal regions of chromosomes, with PUN additionally showing interstitial telomeric sequences (ITSs) in seven chromosome pairs [20–26] (Figs. 1e and 2e). FISH with the *Rex 6* probe yielded dispersed signals in portions of the PEX and PUN genomes; these signals were located in interstitial regions of the largest pairs of chromosomes and diverged from the distribution pattern of the CH (Fig. 3a and b).

## Discussion

Both of the *Podocnemis* species studied herein have a diploid number of 2n = 28, which is extremely low compared to the karyotypes described for other species of turtles and derived karyotypes within Testudines [2, 8, 22, 31]. Reptiles exhibit substantial genomic variation through different organizational levels with a wide range of evolutionary rates [25]. Rearrangements detected by comparative analysis of karyotypes are relatively rare, with a minimal rate of homoplasy. Thus, cytogenetic analysis is extremely important for the systematic study of Podocnemidae.

**Fig. 4:**
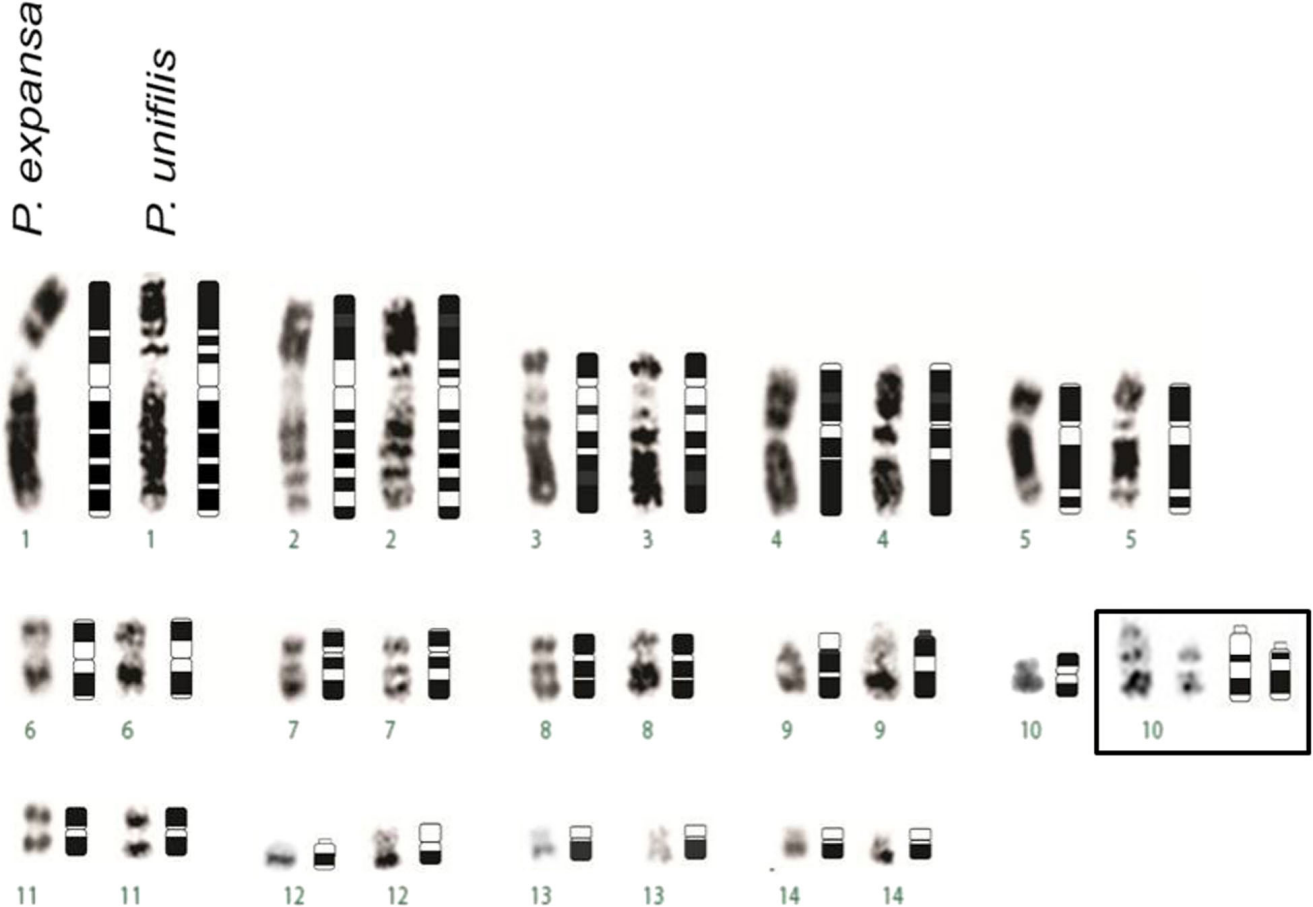
Comparison between *P. expansa* and *P. unifilis* showing G-banding and idiogram. In evidence, pair 10 of PUN that presented size heteromorphism. Bar: 5 μm

According to Rhodin et al. [31], the karyotypes of *P. expansa, P. unifilis, P. lewyana, P. vogli* and *P. sextuberculata* are homogeneous, with karyotypes of 20 metacentric and submetacentric chromosomes, four subtelocentric chromosomes and four acrocentric chromosomes, and FNs of 52. However, G-banding and C-banding subsequently revealed that there were small but classifiable differences in the chromosomes of PEX and PUN (Table 2). Here, we observed the presence of a heteromorphic pair in PUN (pair 10), but we did not observe any heteromorphic sex chromosome. The absence of sexually distinct chromosomes is quite common in this group of vertebrates, whose sexual system is determined by the temperature at which the eggs are incubated [1, 37]. The karyotypic variations observed between PEX and PUN may have arisen via chromosomal rearrangements (e.g., inversions and pericentric heterochromatin duplication), and could form the basis for their interspecific and intraspecific differences (Fig. 4). Gunski et al. [18], in studying PUN, suggested the existence of a pericentric inversion that did not affect heterochromatin in one submetacentric chromosome, as well as a duplication of heterochromatin in a telocentric chromosome. Here, we describe a different and novel chromosomal organization for this species (Table 2). The results of the present study show that pair 13 are submetacentric in both species, while pair 9 is submetacentric in PEX and acrocentric in PUN. These morphologic differences are probably the result of inversion-type rearrangements (Fig. 4). Based on the chromosome evolution approach and cytogenetic banding techniques, we suggest that chromosomal inversion is the main evolutionary strategy in the karyoevolution process, preserving the same diploid value for the Podocnemidae family. Comparative analysis of cytogenetic data between *Podocnemis* species presents a shared karyotype microstructure (2n = 28), indicating little diversification between genders. This work shows a marked degree of chromosomal conservatism for the group.

**Table 2:** Karyotype data reported for PEX and PUN

*M*		*P. unifilis*		References
KF	FN	KF	FN	
20 m/sm + 4st + 4a	52	20 m/sm + 4st + 4a	52	Ayres et al. (1969) [2]
20 m/sm + 4st + 4a	52	20 m/sm + 4st + 4a	52	Huang & Clark (1969) [22]
16 m + 2sm + 10a	54	-	-	Fantin & Monjelió (2011) [13]
22 m + 2sm + 2 t + 2a	54	22 m + 4 t + 2a	52	Gunski et al. (2013) [18]
24 m/sm + 2st + 2a	54	22 m/sm + 2st + 2a	52	Present work

**Fig. 5:**
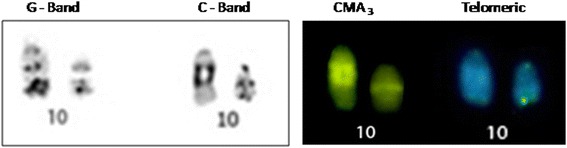
Heteromorphism of pair 10 of *P. unifilis*, confirmed by amplification of GC-rich sequences in the interstitial region of one of the homologues. Bar: 5 μm

The heteromorphism found in pair 10 of PUN is characterized by CH amplification in one of the homologs (Fig. 5), perhaps due to unequal crossing-over, transpositions, and/or regional duplications. CH can be considered a distinctive species-level marker and may even vary between individuals of the same species [39], meaning that the identification of the sites, sizes and DNA compositions of CH is essential for the chromosomal characterization of an organism. In *Podocnemis vogli*, Ortiz et al. [28] described CH in the centromeric, pericentromeric and telomeric regions of several chromosomes. Here, we report that the distribution of CH is quite different between PEX and PUN. Changes of CH are very fast in isolated populations, these changes can lead to speciation [23]. CH is an important marker in the evolutionary context, we suggest, therefore, that the difference in heterochromatin between PUN and PEX could be caused by a recent amplification process in PUN due to reproductive isolation since the species have very similar karyotypes.

Our finding that the NOR is located on the first chromosome pair of both PEX and PUN, associated with a secondary constriction, is consistent with previous observations in *P. expansa* and *P. sextuberculata* [13, 42]. This suggests that the NORs are highly conserved in family Podocnemidae. Similar results were also obtained in the turtle *Macrochelys temminckii* (2n = 52) of the Chelydridae family [7]. Although the number of NORs is conserved in turtles, the NORs may be highly variable in size and chromosomal location. This suggests that duplications and/or deletions have occurred during the evolution of these animals [5, 36].

Reptile chromosomal studies revealed that turtles and crocodiles tend to accumulate and preserve repetitive DNAs as telomeric sequences, satellite DNAs, centromeres and transposons, including those with unknown gene function. Studies in SINES sequences contributed to the phylogeny of various vertebrates including turtles, which grouped Testunididae with Bataguridae [32]. No molecular cytogenetic analysis of the *Podocnemis* genus has been developed so far. To enhance knowledge of the group phylogeny, hypotheses must be tested to verify the importance of repetitive sequences in the composition and structure of chromosomes in turtles. This would improve understanding of the evolution within the family.

**Fig. 6:**
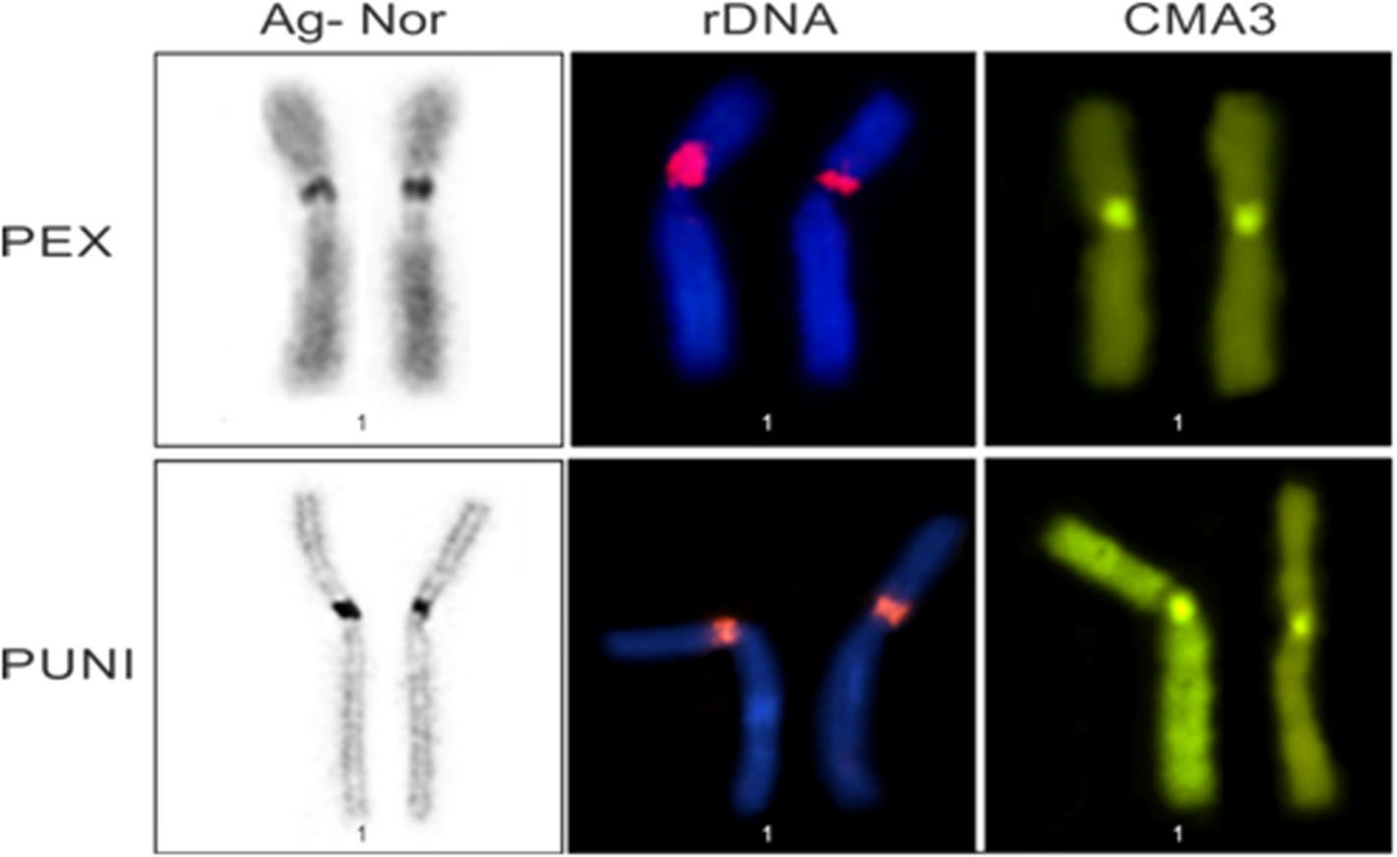
Comparison between *P. expansa* and *P. unifilis* showing NOR, rDNA and CMA3 staining. Bar: 5 μm

This is the first FISH-based molecular cytogenetic analysis of PEX and PUN. The 18S rDNA probes yielded signals that coincided with the NORs and flanked the CMA3-stained regions, indicating that this region is GC-rich (Fig. 6). In contrast, Ventura et al. [42] reported that the secondary constriction region of *Peltocephalus dumerilianus* presented a large block of heterochromatin. The telomeric probes hybridized with atypical regions, such as centromeres, perhaps reflecting the random amplification of telomere-like sequences or telomere remnants that remained following centric fusions that occurred during evolution [10, 14, 43]. ITSs were found in PUN but not PEX; this may reflect that PUN (but not PEX) lost the organization of its (TTAGGG)n sequences, giving PUN an autapomorphic characteristic within the genus.

In both PEX and PUN, FISH revealed that the *Rex 6* TEs were densely distributed in the largest chromosome pairs, more dispersed in euchromatic regions, and highly congregated near AT-rich DNA, a few active genes and in CH-poor regions. This is quite different from the organization in Cichlids, where *Rex 6* occurs in clusters that correlate with the structure and organization of heterochromatic areas [40]. Conversely, in *Erythrinus erythrinus*, *Rex 6* presents a dispersed pattern that is similar to our results [45]. The movement of these TEs can produce structural changes. This, in turn, may trigger chromosomal rearrangements, modifications in gene regulation patterns, and genetic variability. Additional cytogenetic studies in other species of genus *Podocnemis* are needed to further elucidate the karyotypic evolution and rearrangements (inversions, translocations) that have contributed to the differentiation of this group.

Chalopin et al. [11], presenting an overview of the content, diversity and evolution of transposable elements (TEs) in some vertebrate lineages, noted that strong genomic divergence during the evolutionary process of vertebrates may arise from the activities of TEs, which could potentially contribute to understand regulation and the acquisition of new gene functions. Improvements in genome sequencing technologies have enabled researchers to identify numerous sequences as potential chromosome-mapping probes. Among them, TEs are particularly useful because their repetitive nature generates easily visible chromosomal signals. Recent studies show diversity in the location of TEs (*Rex 1*, *Rex 3* and *Rex 6*) in fish. The majority of which are distributed in heterochromatic regions, organized in clusters or dispersed, and associated with multigene families (5S). The association of *Rex 3* to 5S rDNA can lead to gene dispersion [17, 40, 45]. *Rex 6* mobility, in euchromatic regions of PUN and PEX, can probably interfere with the gene regulation process of these turtles.

Despite the low diploid number (2n = 28) being considered derived among Testudines, our results confirm the karyotypic stability of the Podocnemidae family. The markers used herein revealed little interspecific variation in *P. expansa* and *P. unifilis. P. expansa* basal phylogenetic position, in relation to *P. unifilis*, based on molecular data, according to Vargas-Ramirez et al. [41], reflects the cytogenetic results, which show the presence of chromosomic heteromorphy through amplification of repetitive sequences like-telomeres in *P. unifilis*. According to Bickham & Carr [6], Chelonia reached the maximum degree of ecological adaptation leading to an evolutive stationary situation. The karyotype differences found here, among species, are strong karyoevolutive markers. They lead to the understanding of genome organization in turtles, corroborating ecological, reproductive and group conservation studies.

## Conclusion

In sum, our comparative analysis of karyotypes suggests that chromosomal rearrangements are responsible for the differences in karyotype formula (but not the diploid number) between the two studied turtle species. We describe a novel chromosomal organization for this species. Based on the chromosome evolution approach and cytogenetic banding techniques, we suggest that chromosomal inversion is the main evolutionary strategy in the karyoevolution process, preserving the same diploid value for the Podocnemidae family. We, therefore, propose that *Rex 6* may have played a fundamental role in shaping the gene evolution and genomic structure of Chelonia due to its distribution in euchromatic regions and the possibility of interfering in gene regulation. This first description of *Rex 6* in the genome of *Podocnemis* improves our understanding of the dynamics and architecture of these elements, and their contribution to the evolutionary genomics of turtles.

## Methods

### Animal sampling and basic cytogenetic analysis

Six specimens of PEX and four specimens of PUN were collected from Museu Paraense Emílio Goeldi and Bosque Rodrigues Alves in Belém/PA, Brazil. Chromosomes were obtained from two separate methods: peripheral blood lymphocytes and cultured according to the procedure described by Moorhead et al. [27] and primary fibroblasts were cultured following the protocols of Heald et al. [20]. Chromosome spreads were analyzed by G-banding [35], C-banding [38], Ag-NOR staining [21] and CMA_3_ staining [34].

### Chromosome probes and fluorescence in situ hybridization

Fluorescence in situ hybridization (FISH) was performed using the following: 18S rDNA probes from *Prochilodus argenteus* [19], the probes were labeled with biotin or digoxigenin by nick translation and detected with avidin-CY3 or anti-digoxigenin-FITC [26]; human telomeric probes (TTAGGG)n prepared according to the ONCOR protocol; and probes for *Rex 6* were PCR-amplified (forward, 5′ TAAAGCATACATGGAGCGCCAC 3′ and reverse, 5′ GGTCCTCTACCAGAGGCCTGGG 3′) as previously described by Volff et al. [44]. The *Rex 6* PCR products were cloned into pGEM-T plasmids (Promega) and used to transform DH5α (Invitrogen) *Escherichia coli* competent cells. Positive clones were sequenced with an ABI Prism 3100 automatic DNA sequencer (Applied Biosystems) using a Dynamic Terminator Cycle Sequencing kit (Applied Biosystems). The consensus sequences were deposited in the GenBank database (accession numbers KR336815 - KR336823).

## Abbreviations

CH: Constitutive heterochromatin
CMA3: Fluorochrome, Chromomycin A3
FISH: Fluorescence in situ hybridization
FNs: Fundamental numbers
ITSs: Interstitial telomeric sequences
KFs: Karyotypic formulas
NORs: Nucleolar organizing regions
PEX: *Podocnemis expansa*
PUN: *Podocnemis unifilis*
TE: Transposable element

## References

[1] Alho CJR, Danni TMS, Padua LFM (1984). Influência da temperatura de incubação na determinação do sexo da tartaruga da Amazônia *Podocnemisexpansa* (Testudinata, Pelomedusidae). Rev Bras Biol.

[2] Ayres M, Sampaio MM, Barros RMS, Dias LB, Cunha OR (1969). A karyological study of turtles from the Brazilian Amazon Region. Cytogenetics.

[3] Barros RM, Sampaio MM, Assis MF, Ayres M (1976). General considerations on the karyotypic evolution of Cheloniidae from the Amazon region of Brazil. Cytologia.

[4] Bérnils RS and Costa HC Brazilian reptiles: List of species. Version 2012.1.

[5] Bickham JW (1981). Two-hundred-million-year-old chromosomes: deceleration of the rate of karyotypic evolution in turtles. Science.

[6] Bickham JW, Carr JL (1983). Taxonomy and phylogeny of the higher categories of cryptodiran turtles based on a cladistics analysis of chromosomal data. Copeia.

[7] Bickham JW, Rogers DS (1985). Structure and variation of the nucleolus organizer region in turtles. Genetica.

[8] Bull JJ, Legler JM (1980). Karyotypes of side necked turtles (Testudines, Pleurodira). Can J Zool.

[9] Cardoso DC (2014). das Graças Pompolo S, Cristiano MP, Tavares MG. The Role of Fusion in Ant Chromosome Evolution: Insights from Cytogenetic Analysis Using a Molecular Phylogenetic Approach in the Genus Mycetophylax. PLoS One.

[10] Castiglia R, Garagna S, Merico V, Oguge N, Corti M (2006). Cytogenetics of a new cytotype of African *Mus* (subgenus Nannnomys) *minutoides* (Rodentia, Muridae) from Kenya: C– and G–banding and distribution of (TTAGGG)n telomeric sequences. Chromosome Res.

[11] Chalopin D, Naville M, Plard F, Galiana D, Volff JN (2015). Comparative analysis of transposable elements highlights mobilome diversity and evolution in vertebrates. Genome Biol Evol.

[12] Ernst CH, Barbour RW (1989). Turtles of the World.

[13] Fantin C, Monjeló LAS (2011). Cytogenetic studies in *Podocnemis expansa* and *Podocnemis sextuberculata* (Testudines, Podocnemididae), turtles of the Brazilian Amazon. Caryologia.

[14] Faravelli M, Azzalin CM, Bertoni L, Chernova O, Attolini C, Mondello C, Giulotto E (2002). Molecular organization of internal telomeric sequences in Chinese hamster chromosomes. Gene.

[15] Ferri V (2002). Turtles & Tortoises: A Firefly Guide.

[16] Feschotte C, Pritham EJ (2007). DNA transposons and the evolution of eukaryotic genomes. Comp Cytogenet. Annu Rev Genet..

[17] Gross MC, Schneider CH, Valente GT, Porto JIR, Martins C, Feldberg E (2009). Comparative cytogenetic analysis of the genus Symphysodon (discus fishes, Cichlidae): chromosomal characteristics of retrotransposons and minor ribosomal DNA. Cytogenet Genome Res.

[18] Gunski RJ, Cunha IS, Degrandi TM, Ledesma M, Garnero ADV (2013). Cytogenetic comparison of *Podocnemis expansa* and *Podocnemis unifilis*: a case of inversion and duplication involving constitutive heterochromatin. Genet Mol Biol.

[19] Hatanaka T, Galetti PM (2004). Mapping of the 18S and 5S ribosomal RNA genes in the fish *Prochilodus argenteus* Agassiz, 1829 (Characiformes, Prochilodontidae). Genetica.

[20] Heald KA, Hall CA, Downing R (1991). Isolation of islets of Langerhans from the weanling pig. Exp Diabetes Res.

[21] Howell WM, Black DA (1980). Controlled silver-staining of nucleolus organizer regions with a protective colloidal developer: a 1–step method. Experientia.

[22] Huang CC, Clark HF (1969). Chromosome studies of the cultured cells of two species of sidenecked turtles (*Podocnemis unililis* and *P. expansa*). Chromosoma.

[23] Hughes SE, Hawley RS (2009). Heterochromatin: A Rapidly Evolving Species Barrier. PLoS Biol.

[24] Iverson JB. A revised checklist with distribution maps of the turtles of the world. Privately printed: Richmond; 1992. p. 363.

[25] Janes DE, Organ CL, Fujita MK, Shedlock AM, Edwards SV (2010). Genome evolution in Reptilia, the sister group of mammals. Annu Rev Genomics Hum Genet.

[26] Martins C, Galetti PM (1998). Karyotype similarity between two sympatric Schizodon fish species (Anostomidae, Characiformes) from the Paraguai River basin. Gen Mol Biol.

[27] Moorhead PS, Nowell PC, Mellman WJ, Battips DM, Hungerford DA (1960). Chromosome preparations of leukocytes cultured from human peripheral blood. Exp Cell Res.

[28] Ortiz ML, Rodríguez PA, Bueno ML. Caracterización citogenética de la tortuga sabanera Podocnemis vogli (reptilia: testudinata: podocnemididae). Acta Biológica Colombiana. 2005;19–33.

[29] Pritchard PCH (1979). Encyclopedia of turtles.

[30] Pritchard PCH, Trebau P. The Turtles of Venezuela, contributions in herpetology. n2. 1984.

[31] Rhodin AJ, Mittermeier AL, Gardner AL, Medem F (1978). Karyotypic Analysis of the *Podocnemis* Turtles. Copeia.

[32] Sasaki T, Takahashi K, Nikaido M, Miura S, Yasukawa Y, Okada N (2004). First application of the SINE (Short Interspersed Repetitive Element) method to infer phylogenetic relationships in reptiles: an example from the turtle superfamily testudinoidea. Mol Biol Evol.

[33] Schneider CH, Gross MC, Terencio ML, Carmo EJ, Martins C, Feldberg E. Evolutionary dynamics of retrotransposable elements *Rex 1*, *Rex 3* and *Rex 6* in neotropical cichlid genomes. BMC Evol Biol. 2013;13:152. 10.1186/1471-2148-13-152 PMC3728117 23865932

[34] Schweizer D (1980). Simultaneous fluorescent staining of R bands and specific heterochromatic regions (DA/DAPI bands) in human chromosomes. Cytogenet Cell Genet.

[35] Seabright M (1971). A rapid banding technique for human chromosomes. Lancet.

[36] Shaffer HB, Meylan P, Mcknight ML (1997). Tests of turtle phylogeny: Molecular, morphological and paleontological approaches. Syst Biol.

[37] Souza RR, Vogt RC (1994). Incubation temperature influences sex and hatchling size in the neotropical turtle *Podocnemis unifilis*. J Herpetol.

[38] Sumner AT (1972). A simple technique for demonstrating centromeric heterochromatin. Exp Cell Res.

[39] Sumner AT (2003). Chromosomes - organization and function.

[40] Valente GT, Mazzuchelli J, Ferreira IA, Poletto AB, Fantinatti BEA, Martins C. Cytogenetic Mapping of the retroelements *Rex 1*, *Rex 3* and *Rex 6* among cichlid fish: new insights on the chromosomal distribution of transposable elements. Cytogenet Genome Res. 2011;133:34–42. 10.1159/000322888 21196713

[41] Vargas-Ramíreza M, Castaño-Morab OV, Fritza U (2008). Molecular phylogeny and divergence times of ancient South American and Malagasy river turtles (Testudines: Pleurodira: Podocnemididae). Org Divers Evol.

[42] Ventura K, Moreira CN, Moretti R, Yonenag-Yassuda Y, Rodrigues MT (2014). The lowest diploid number in Testudines: Banding patterns, telomeric and 45S rDNA FISH in Peltocephalus dumerilianus, 2n = 26 and FN = 52 (Pleurodira, Podocnemididae). Genet Mol Biol.

[43] Vermeesch JR, De Meurichy W, Van Den Berghe H, Makynen P, Petit P (1996). Differences in the distribution and nature of the interstitial telomeric (TTAGGG)n sequences in the chromosomes of the Giraffidae, okapai. (*Okapia johnstoni*), and giraffe (*Giraffa camelopardalis*): evidence for ancestral telomeres at the okapi polymorphic rob(4: 26) fusion site. Cytogenet Cell Genet.

[44] Volff JN, Körting C, Froschauer A, Sweeney K, Schartl M (2001). Non-LTR retrotransposons encoding a restriction enzyme-like endonuclease in vertebrates. J Mol Evol.

[45] Yano CF, Bertollo LAC, Molina WF, Liehr T, Cioffi MB (2014). Genomic organization of repetitive DNAs and its implications for male karyotype and the neo-Y chromosome differentiation in *Erythrinus erythrinus* (Characiformes, Erythrinidae). Comp Cytogenet.

